# Recent Advances in the Prediction of Subcellular Localization of Proteins and Related Topics

**DOI:** 10.3389/fbinf.2022.910531

**Published:** 2022-05-19

**Authors:** Kenta Nakai, Leyi Wei

**Affiliations:** ^1^ Institute of Medical Science, The University of Tokyo, Minato-Ku, Japan; ^2^ School of Software, Shandong University, Jinan, China

**Keywords:** subcellular localization, deep learning, subcellular proteomics, amino acid sequence analysis, image analysis

## Abstract

Prediction of subcellular localization of proteins from their amino acid sequences has a long history in bioinformatics and is still actively developing, incorporating the latest advances in machine learning and proteomics. Notably, deep learning-based methods for natural language processing have made great contributions. Here, we review recent advances in the field as well as its related fields, such as subcellular proteomics and the prediction/recognition of subcellular localization from image data.

## Introduction

In bioinformatics, prediction of subcellular localization sites of proteins from their amino acid sequences has remained to be an important field. Such studies are useful in understanding the mechanisms of their localization process, including the recognition of their sequence determinants (i.e., the sorting/targeting signals) coded in amino acid sequences, and in inferring the function of those proteins. The prediction problem has also been used as a benchmark place for introducing latest machine-learning algorithms. Many review articles including ours have been published in this field. In this review, we would like to introduce recent advances mainly published after our latest review (Imai & Nakai, 2020). We will also briefly review recent papers in related areas, such as the prediction/recognition of protein subcellular localization based on image data and the subcellular proteomics, which seems to give us hints for the future direction of this field.

## General Reviews and Assessment Studies

Several general review articles have been published recently (Nielsen, Konstantinos, et al., 2019; Kumar & Dhanda, 2020; Barberis et al., 2021; Jiang, Wang, Wang, et al., 2021; Pan et al., 2021). Of these, the review by Nielsen et al. is a retrospective of the field, emphasizing the prediction of signal peptides; Kumar and Dhanda give a full list of available tools and their classification; and the review by Jiang et al. emphasizes the mathematical foundation of various approaches, also giving a review of some benchmark datasets. Similarly, Shen et al. reports their critical evaluation of web-based tools for protein subcellular localization (Shen et al., 2020), though they seem to mainly focus on Gene Ontology-based predictors, which are basically out of the scope of this review. Although the prediction of subcellular localization of RNA molecules is also out of the scope, it is important to establish reliable data sources for training the prediction models (Cui et al., 2022) and how to integrate heterologous resources is an important issue (Savulescu et al., 2021).

As for the prediction of specific sorting signals, two assessment studies on the prediction of signal peptides based on experimental data of rather untypical organisms were recently published: these organisms are phytoplasmas (Garcion et al., 2021) and a thermoacidophilic archaeon (Singhal et al., 2021). It seems that the current predictors are not highly reliable for the prediction of these organisms (probably due to the scarcity of their training data); among several versions of SignalP (see below), not the latest version was the most reliable. We believe that important future issues in the prediction of signal peptides are 1) the improvement of distinction between cleavable signal peptides and uncleaved N-terminal transmembrane segments and 2) the prediction of secretory proteins without (apparent) signal peptides (Lonsdale et al., 2016; Nielsen, Petsalaki, et al., 2019).

## Deep Learning and Language Model-Based Methods

As noted above, the prediction of protein subcellular localization has always been a playground where the latest machine learning algorithms are introduced. In recent years, deep learning-based methods have become quite popular and thus a number of papers have been published within a few years (Cong et al., 2020, 2022; Semwal & Varadwaj, 2020; Jiang, Wang, Yao, et al., 2021; Liao et al., 2021; Yuan et al., 2021). The architecture of deep learning models has made rapid progress and they have also been applied to bioinformatics, such as protein design (Ding et al., 2022). Convolutional neural networks (CNNs) are the standard model; Liao et al. introduced the PSSMs (position-specific scoring matrices) derived from PSI-BLAST (Altschul et al., 1997) for adding evolutionary information to input; Cong et al. used the ant-colony optimization for letting the prediction model self-evolving (Cong et al., 2020), which is a trend of deep learning. As another big trend, techniques that have been successfully used in natural language processing have been introduced. One of them is the use of (multi-head) self-attention mechanism, which was first introduced in Transformer (reviewed in Shreyashree et al., 2022). Both Jiang et al. and Cong et al. report the improvement of prediction performance with the use of the self-attention mechanism (Jiang, Wang, Yao, et al., 2021; Cong et al., 2022). Jiang et al. also claim that their method shows better performance in suborganellar prediction (see below).

Deep neural networks have also been used in the prediction of sorting signals, particularly signal peptides (J.-M. Wu et al., 2019). SignalP, a standard tool for signal-peptide prediction, has employed this technique since its version 5 (Almagro Armenteros et al., 2019). The same group also used a similar method, incorporating the self-attention mechanism, for TargetP, which can detect three kinds of targeting signals (Almagro Armenteros et al., 2019). One of the merits of using attention-based methods is that it enables us to see which parts of the input sequence are paid with greater “attention”. Thus, Almagro Armenteros et al. found that the second amino acid, which appears after the first methionine, seems to be important for the recognition of targeting signals, such as the chloroplast transit peptides. Wu et al. used their Transformer-based model for a different purpose: they generated novel signal peptides from the model that learned a number of known signal peptides in many organisms (Z. Wu et al., 2020). They confirmed experimentally that the generated peptides actually worked as functional signal peptides when appended to the N-terminus of cytosolic proteins in *Bacillus subtilis*, though these sequences were not similar to any of known ones.

After the appearance of Transformer, a kind of its successor model, BERT (Bidirectional Encoder Representations from Transformers), has become very popular in natural language processing. One of the characteristics of BERT is that the model has been pretrained with a very large unannotated (unlabled) training set and that users are usually expected to fine-tune the model with their own labeled data. This style, called the transfer learning, meaning that pre-trained results are transferred to specific topics, has become a new trend even in molecular biology, too. Heinzinger et al. used this approach and fine-tuned their model as an example, for the prediction of protein subcellular localization sites (Heinzinger et al., 2019). Jin et al. also applied their pre-trained model to the subcellular localization prediction (Jin & Yang, 2022). Nowadays, models pretrained with both DNA (Ji et al., 2021) and amino acid sequences, ProtTrans (Elnaggar et al., 2021), are available for end users. Indeed, SignalP ver. Six was constructed based on ProtTrans and a significant improvement of its performance is reported (Teufel et al., 2022).

The multi-head self-attention model with multi-scale (i.e., parallel for various scales) CNNs was also used for the prediction of subcellular localization sites of mRNAs (D. Wang et al., 2021). Their system not only predicts multiple localizations of mRNA isoforms but also is useful in interpreting the mechanisms/signals of isoform-specific localization, based on the analysis of attention weights.

## Miscellaneous Algorithms

Of course, prediction methods which do not rely on deep learning but on other machine learning methods have also been published recently in this field. Most of them use general sequence features rather than hand-crafted features related to specific sorting signals and claim to be able to address the problem of proteins localized at multiple sites (i.e., multi-labeled proteins), though there still remains the problem that their training data do not seem to have been annotated with a uniform criterion (see below). Some of them proposed extensions of existing sequence features, such as the *k*-mer compositions (Li et al., 2019; Yao et al., 2019; Sahu et al., 2020), while some imported external information, such as Gene Ontology and protein-protein interactions (Chen et al., 2021; Liu et al., 2021; Zhang et al., 2021). One method employed an ensemble approach of multiple classifiers with voting, claiming that the approach is effective in addressing the problem of imbalanced sizes of training data between different localization sites (Wattanapornprom et al., 2021). Among these newly published reports, the approach reported by Alaa et al. might be a bit novel and have further room for improvements: they used Markov models to produce a feature vector which is based on the micro-similarities of the probability distributions between the input sequence and the reference models (Alaa et al., 2019). Moreover, it might be notable that Wang et al. attempted to incorporate hand-crafted features derived from the three-dimensional structure information of input proteins, such as their substructure frequency (G. Wang et al., 2021). Although the improvement of performance may not look so striking in their analysis, this field would be promising because of the current significant improvement of the prediction accuracy in the three-dimensional structure of proteins through AlphaFold2 and related methods (Jumper et al., 2021). It seems that the 3D structure-based approach could be promising in understanding how some sorting signals that do not show apparent sequence similarity are specifically recognized. In addition, it has been known that there are some correlations between the pI value of proteins and their subcellular localization [reviewed in (Tokmakov et al., 2021)], which might be the reason why amino acid composition is an effective feature for the prediction. Comprehensive analyses using predicted structures would be useful in understanding the adaption of individual proteins to the environment provided by each localization site.

## Prediction of Localization at Specific Organelles and Suborganellar Localization

Probably, partly because of a certain level of maturation in the prediction of protein subcellular localization and partly because of the progress of precise subcellular proteomics studies (see below), it seems that a boom in the prediction of suborganellar localization has come. Its most typical field is the prediction of submitochondrial localization [for a recent review, see (Martelli et al., 2021)]. In mitochondria, about 1000 proteins encoded in the nuclear genome are sorted into four places (the matrix, the inner membrane, the intermembrane space, and the outer membrane). Several predictors have been released in only a recent few years; most of them were based on (convolutional) deep learning. For example, Savojardo et al. developed DeepMito, which is based on CNN and the method was used to annotate the potential mitochondrial proteins of four species (Savojardo, Bruciaferri, et al., 2020; Savojardo, Martelli, et al., 2020). Wang et al. proposed another predictor (DeepPred-SubMito), which is also based on CNN and was taken care of the unbalanced sample sizes with the random over-sampling approach (X. Wang et al., 2020). Hou et al. developed another predictor (iDeepSubMito), which is based on CNN and the bidirectional LSTM (i.e., Long Short Term Memory) with the self-attention model (Hou et al., 2021). In contrast, Yu et al. developed a predictor (SubMito-XGBoost), which is based on eXtreme gradient boosting, a new method in traditional machine learning, combining gradient boosting and random forest ensemble learning (Yu et al., 2020). Unlike CNN-based methods, this method uses a variety of sequence features, such as the gapped dipeptide composition. It seems possible that such approaches will be useful in finding hidden sorting signals. More explicitly, Schneider et al. developed a specialized predictor (iMLP) for detecting internal matrix targeting-like sequences, which do not exist on the N-terminus, unlike well-known mitochondrial (matrix) targeting peptides, using recurrent neural networks (RNNs) (Schneider et al., 2021).

Besides mitochondria, recent works on the prediction of suborganellar localization are not many. One for subnuclear localization (L. Wu et al., 2020), another for sub-peroxisomal localization (Anteghini et al., 2021), and another for distinguishing *cis*-Golgi and *trans*-Golgi proteins using deep learned 107 features (Lv et al., 2021). However, we believe that these works become pioneers for subsequent more elaborated works. In addition, there are a few works dealing with some specialized but biologically meaningful problems: Kaundal et al. proposed two-step predictions, where input proteins are classified into plastid or non-plastid proteins in the first step and the plastid proteins are further classified into one of the four types chloroplasts, chromoplasts, etioplasts, and amyloplasts) (Kaundal et al., 2013). Kaleel et al. presented a CNN-based predictor which simply detects endomembrane system and secretory pathway proteins from the others (Kaleel et al., 2020). It would be interesting to see how such an approach could complement traditional approaches based on the recognition of signal peptides.

At the end of this section, where a new trend in this field is introduced, we would like to add one paper, which may become a pioneering work in a new trend: prediction of tissue-specific subcellular localization of proteins. Zhu et al. attempted this based on the information of tissue-specific functional associations and protein-protein interactions (PPIs) (Zhu et al., 2019). They identified 1314 known differential localizations between nine types of tissues as well as 549 novel candidates, some of which were verified through literature survey. With the increase of more known examples, further approaches should be taken to improve the prediction accuracy.

## Localization of Bacterial Proteins

As far as we notice, for the prediction of the subcellular localization of bacterial proteins, only a few new predictors have been released after the publication of our previous review (Imai & Nakai, 2020), where we already reviewed PSORTm for the prediction for metagenome data (Peabody et al., 2020) and PSORTdb 4.0, which contains both experimentally-verified and computationally-predicted subcellular localization information (Lau et al., 2021), for example. We have also mentioned PSO-LocBact, which gives a kind of consensus between various existing predictors using the particle swarm optimization (PSO) algorithm (Lertampaiporn et al., 2019). However, we noticed a few new works in more specific prediction problems: GP4 is a predictor specifically developed for the prediction of Gram-positive proteins (Grasso et al., 2021). Next, T3SEpp is a predictor for bacterial type III secreted effectors (T3SEs), which are used for the infection of Gram-negative bacteria, and thus the prediction has medical importance (Hui et al., 2020). Finally, BetAware-Deep is a web server for the discrimination and topology prediction of prokaryotic transmembrane beta-barrel proteins (Madeo et al., 2021): this discrimination is useful for the prediction of subcellular localization because these beta-barrel proteins exist in the outer membrane of Gram-negative bacteria. In addition, the topology prediction could be also useful for further predictions because it is important to know that an additional signal faces to which side of the membrane (i.e., the periplasm and the outside of the cell).

## Subcellular Proteomics

For the development of reliable prediction methods, comprehensive lists of known (i.e., experimentally-verified) localization information are essential. Although such information is contained in standard databases, such as UniProt/Swiss-Prot (Bateman et al., 2021), it is desirable that the localization is determined in a uniform criterion because information that a protein is localized at a single site or multiple sites can be sometimes ambiguous, for example, and thus it must be decided in an objective manner. Recently, there have been notable advances in subcellular proteomics and thus we briefly introduce these advances in this section, hoping that these trends could be indicators for the future direction of novel prediction methods.

In the field of transcriptomics, obtaining the expression profile of individual cells (single cell RNA sequencing, scRNA-seq) has become rather popular. With such approaches, we can now explore what kind of cell types are contained in a certain tissue. Moreover, the techniques to clarify the spatial distribution of these cell types (spatial transcriptomics) is under rapid development. And the information of subcellular distribution of RNAs has begun to accumulate, too (Longo et al., 2021). This field will undoubtedly stimulate the further development of methods for the prediction of RNA subcellular localization.

In the field of proteomics, equivalent studies exist. For example, the importance of single-cell proteomics is increasing because the amount of mRNAs is not enough to know the amount of their protein products (Xie & Ding, 2022). However, it seems that currently the word, spatial proteomics, means subcellular proteomics, reflecting the current excitement in this field. Naturally, many review articles have been published in only a few years (Lundberg & Borner, 2019; Borner, 2020; Christopher et al., 2021, Christopher et al., 2022; Paul et al., 2021). Amongst them, Christopher et al. (2022) compares the methods used in subcellular transcriptomics and proteomics.

There are several approaches in subcellular proteomics and they can be classified in various ways. Perhaps, the most standard method is to directly observe the localization of a protein labeled by fluorescent antibody (or any other affinity reagents) through microscopic imaging *in situ*. The analysis of the obtained images requires computational procedures, where machine learning has made significant contributions (see the next section). The Subcellular section of the Human Protein Atlas (HPA) database contains the immunofluorescence images of 65% of the human protein-coding genes (Thul et al., 2017). Based on this collection, a competition for machine-learning-based image processing methods (the Kaggle competition for multi-label classification of cell organelles in proteome scale Human Protein Atlas data) was held (Ouyang et al., 2019).

Other direct methods include the physical separation of specific organelles and the biochemical fractionation of cells using centrifugation or detergents. Proteins contained/enriched in the separated organelle or the specific fraction are determined using tandem mass spectroscopy (MS). Since proteins that are co-localized at the same compartment should share the same distribution of their abundance between fractions, computational methods, such as the cluster analysis (known as correlation profiling), can be used to identify the subcellular localization of thousands of proteins simultaneously (Itzhak et al., 2019; Borner, 2020). If fractions are labeled with different isotope tags, like in the LOPIT (Localization of Organelle Proteins by Isotope Tagging) method, more accurate distinction can be made between organelles with similar densities (such as Golgi, plasma membrane and endoplasmic reticulum) (Elzek et al., 2021). Using an MS-based pipeline, Orre et al. identified the subcellular localization of about 12,000 proteins across five cell lines and the data are available as SubCellBarCode (Orre et al., 2019). It is interesting to see what kind of factors contribute to differential localization across cell types. Joshi et al. determined the three types of localization (cytosolic, nuclear, and membrane) of 6572 proteins in human T cells. They also monitored the time-course changes after the T cell receptor stimulation and identified about 200 potentially translocating proteins (Joshi et al., 2019). The data are released as TCellSubC. Huang et al. also constructed a database (PSL-LCCL) of protein subcellular localization (six organelles) in human cancer cell lines (Huang et al., 2022). Finally, a database of mitochondrial proteome (MitoCarta) was made mainly from an MS-based approach and its latest version contains the sub-organellar localization information (Rath et al., 2021). These resources could be useful in planning the future directions of subcellular localization predictions. It might be noteworthy that the advances in MS technology have enabled the peptidome and metabolome analyses in the single-cell level (Nemes, 2021).

The third systematic approach is to detect the protein-protein interaction network (Pino & Schilling, 2021). The detection can be made with co-immunoprecipitation or cross-linking. Recently, an approach called proximity labeling is increasingly used, where an expression plasmid containing the gene of a bait protein fused with an engineered protein, such as BioID (biotin ligase) or APEX (ascorbic acid peroxidase), is introduced into target cells; after the fused protein is expressed within the cells, the addition of biotin etc. causes labeling reactions, where nearby proteins are biotinylated; then the biotinylated proteins are collected and identified through LC-MS (Liquid Chromatography Mass Spectrometry). With this approach, no specific antibodies are required. Notably, a large BioID-based map of human cells (HEK293) was published recently (Go et al., 2021). In this work, the authors defined the intracellular locations of 4,145 proteins, using 192 subcellular markers. The data are provided at humancellmap.org. This approach seems to be superior in its sensitivity but it does not seem to be good at identifying proteins with multiple locations. Another group also identified the protein proximity network in mitochondria, comprising 1,465 proteins (Antonicka et al., 2020).

## Image Analysis

As compared to amino acid sequences, images that present proteins or subcellular locations with distinct patterns are more intuitive and interpretable. Benefitting from the advent of microscopic imaging techniques, there has been an increasing interest in protein subcellular localization based on the analyses of fluorescence microscope images and immunohistochemistry (IHC) images. The image-based protein subcellular localization methods can be roughly categorized into two groups, i.e., traditional machine learning methods, and deep learning methods. Traditional machine learning methods mainly rely on the design of hand-crafted image feature descriptors for predictive model construction. Typical image features in image processing field, such as Haralick features, Zernike features, and Local Binary Patterns (LBP), etc., are commonly used (Xu et al., 2018). Considering the microscope images are quite different from natural images, directly using the features are not sufficient to capture the key information in microscope images. Therefore, a number of methods based on essential properties of microscope images have been developed. Yang et al. proposed a frequency feature and intensity coding strategy to explore the local region information, improving feature representation of IHC images (Yang et al., 2019). Tahir et al. designed the threshold calculation LBP operator for feature extraction from fluorescence microscopy images (Tahir & Idris, 2020). To analyze the feature patterns from images of multi-location proteins, Xu et al. built the LDA models to extract various feature topics of IHC images and map them to subcellular locations (Xu et al., 2020). However, these hand-crafted image features are shallow and low-level, and cannot fully explore the specificity of different locations, due to the limited knowledge of imaging, thereby impacting the performance in protein subcellular localization.

Recently, deep learning has made breakthroughs in computer vision and has attracted considerable attention in biomedical image analysis, due to its excellent ability in learning high-latent image feature representations (Fuyong et al., 2018). Therefore, recent computational efforts in this field are more focused on deep learning methods. Convolutional neural networks are the very first deep networks that are introduced as the image feature extractor to capture the features for protein subcellular localization (Pärnamaa & Parts, 2017). The deep features learnt from deep neural networks are reportedly to be better than the hand-crafted features (Su et al., 2021; F.Wang & Wei, 2022). Considering the feature space learnt from CNNs may exist redundant information, Su et al. proposed a feature selection strategy to optimize the feature space, thereby improving the predictive performance and meanwhile reducing the computational complexity (Su et al., 2021). Similarly, Long et al. introduced the self-attention mechanism to capture the key features derived from the deep convolutional neural network (Long et al., 2020). Xue et al. combined multiple nonlinear decomposing algorithms to unmix effective feature patterns from deep image feature representations (M.-Q. Xue et al., 2021). To further improve the predictive performance, Hu and colleagues employed a label-correlation relevancy strategy to enhance localization results (Hu et al., 2022). More recently, Wang et al. proposed a multi-scale feature representation learning framework and successfully learnt a set of comprehensive features from low-level to high-level (F. Wang & Wei, 2022). They demonstrate that the deep features from different scales are complementary and useful to capture the distinguishable information amongst different subcellular locations. In addition, some methods integrating deep features with traditional hand-crafted features are proposed. Xue et al. split images into representative patches as model inputs, and integrate feature engineering and deep learning methods (Z.-Z. Xue et al., 2020). UIIah et al. extracted different handcrafted and deep features learned from different viewpoints of the images (Ullah et al., 2021). However, some existing methods still suffer from data imbalance and insufficient data problems. To copy with the issues, Tu et al. proposed a self-supervised learning framework namely SIFLoc through introducing a hybrid data augmentation strategy and contrastive learning (Tu et al., 2022).

Furthermore, these protein subcellular location prediction methods have been used in location biomarker analysis (Fan et al., 2021; Long et al., 2020; F.; Wang & Wei, 2022; Xu et al., 2020; Z.-Z.; Xue et al., 2020). Proteins in the normal and cancerous images are likely to have different subcellular patterns. The methods based on images were expected to detect the differences. To assess the significance of location changes, an independent sample t-test is used to obtain the *p* values for the prediction results of the normal and cancerous images. Through the consistency evaluation analysis between HPA and Swiss-Prot, Xu et al. recently demonstrate that proteins having highly variable locations are more likely to be biomarkers of diseases (Xu et al., 2021). Thus, comprehensive analyses using microscopic images would be useful in speeding up the understanding of the mechanism of protein mislocalization and providing the accurate identification of cancer biomarkers.

## Future Directions and Concluding Remarks

As reviewed here, in only a few years, there have been many advances in this field. Deep learning-based methods will continue to play important roles in both image and sequence analyses. Moreover, since subcellular proteomics-based localization information has become increasingly popular, the need to predict the localization of unknown proteins computationally is becoming less important; rather, we believe that computational approaches should become even more important in the following aspects: 1) to assist the improvement of proteome-based experiments with the use of more sophisticated methods in their data analysis; 2) to contribute to understand specific molecular mechanisms of protein sorting, through the interpretation of learned features (such as the attentions), etc.; 3) to contribute to the understanding of “exceptional” mechanisms of protein sorting, such as the secretion without N-terminal signal peptides (Nielsen, Petsalaki, et al., 2019); 4) to characterize the dynamic translocation processes upon external stimulation, disease, etc.; this includes the effort to interpret these phenomena through the change of their sequences (*via* alternative splicing) or environments. Lastly, 5) the contribution to synthetic biology, e.g., the design of novel targeting signals with desired characteristics, would become more important (Rajendran et al., 2010). The next few years will continue to be exciting for researchers in the field. Almagro Armenteros et al., 2019,Jiang et al., 2021, Nielsen et al., 2019, Savojardo et al., 2020, Wang et al., 2021, Wu et al., 2020.
